# On-chip analysis of glycolysis and mitochondrial respiration in human induced pluripotent stem cells

**DOI:** 10.1016/j.mtbio.2022.100475

**Published:** 2022-10-31

**Authors:** Stefanie Fuchs, Ruben W.J. van Helden, Maury Wiendels, Mees N.S. de Graaf, Valeria V. Orlova, Christine L. Mummery, Berend J. van Meer, Torsten Mayr

**Affiliations:** a Institute of Analytical Chemistry and Food Chemistry, Graz University of Technology, Graz, Austria; b Department of Anatomy & Embryology, Leiden University Medical Center, Leiden, Netherlands

**Keywords:** Extracellular flux, Human stem cells, Sensors, Metabolism, Organ-on-Chip, Oxygen, pH

## Abstract

Recent advances in microfluidic engineering allow the creation of microenvironments in which human cells can be cultured under (patho-)physiological conditions with greater reality than standard plastic tissue culture plates. Microfluidic devices, also called Organs-on-Chip (OoC), allow complex engineering of the cellular compartment, yielding designs in which microfluidic flow can be precisely controlled. However, it is important that cellular physiology is not only controlled but can also be monitored in these devices. Here, we integrated oxygen and pH sensors into microfluidics, allowing close monitoring of the extracellular flux from the cells, enabling constant assessment of features such as glycolysis and mitochondrial oxidative phosphorylation *in situ*. Using human-induced pluripotent stem cells (hiPSCs) as an exemplar of a highly metabolic and relatively challenging cell type to maintain, we showed that monitoring the extracellular environment allowed rapid optimization of the seeding protocol. Based on the measurements, we implemented earlier and more frequent media refreshment to counteract the rapid acidification and depletion of oxygen. The integrated sensors showed that hiPSCs in the devices exhibited mitochondrial and glycolytic capacity similar to that measured with the Seahorse extracellular flux system, the most widely used standard for these types of assays in conventional cell culture. Under both conditions, hiPSCs showed greater reliance on glycolysis than mitochondrial OXPHOS and the absolute values obtained were similar. These results thus pave the way for the assessment of cell metabolism *in situ* under conditions of fluidic flow with the same precision and relevance as current standard static cell cultures.

## Highlights

• Integrated sensors on a microfluidic chip allow accurate assessment of metabolic flux.
• Monitoring pH and dissolved oxygen in organ-on-chips give insight into the metabolic cellular state.
• Optimization of tissue-seeding in organ-on-chip can be rapidly optimized by monitoring pH and oxygen.

## Graphical abstract

**Figure:**
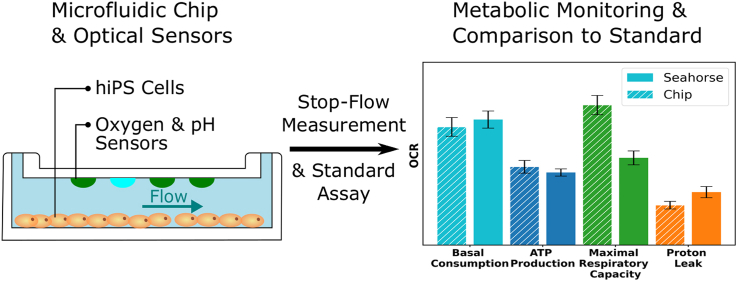

## Abbreviations

Human induced pluripotent stem cells: (hiPSCs)
Organs-on-Chip: (OoC)
Oxygen consumption rate: (OCR)
Extracellular acidification rate: (ECAR)
Carbonyl cyanide-*p*-trifluoromethoxyphenylhydrazone: (FCCP)
2-deoxy-D-glucose: (2-DG)
Oxidative phosphorylation: (OXPHOS)
4-(2-hydroxyethyl)-1-piperazineethanesulfonic acid: (HEPES)
Dulbecco's Modified Eagle Medium: (DMEM)
4-(5,5-difluoro-7-(4-hydroxyphenyl)-1,9-diphenyl-5H-5λ4,6λ4-dipyrrolo[1,2-c: 2′,1′ f][1,3,5,2]triazaborinin-3-yl)-N-dodecylbenzamide: (OHC12)
2-(*N*-morpholino)ethanesulfonic acid: (MES)
Tris(hydroxymethyl)aminomethane: (TRIS)
Platinum(II)meso-tetra(4-fluorophenyl) tetrabenzoporphyrin: (Pt-TPTBPF)

## 1 Introduction

**Fig. A1:**
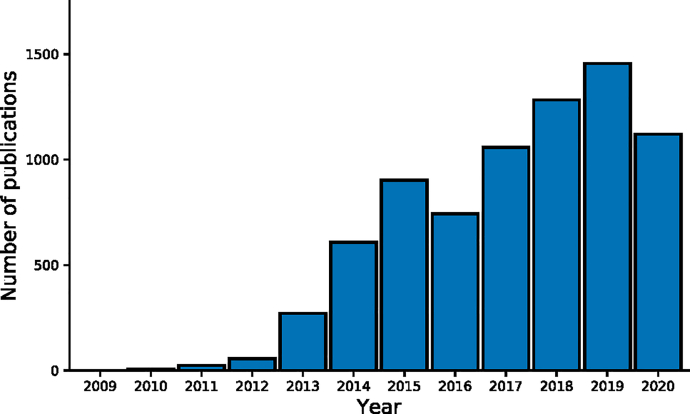
Number of publications per year since the introduction of the Seahorse system [34].

How cells use different metabolites is organ-dependent and can change significantly in different disease states. It is now widely accepted that metabolic shifts can rapidly and profoundly alter cellular behaviour and phenotype by directly regulating the epigenome [[1], [2], [3], [4], [5]]. Parameters like oxygen consumption, changes in pH and glucose consumption can provide information on cellular ATP production, giving insight into the viability and metabolic state of cultured cells. Several commercial assay systems are available to address this in conventional static cell cultures [6,7]. One of the most widely used systems is the Agilent XF Analyzer, known as the Seahorse™ [6]. Since its launch in 2006, more than 5000 publications have reported data on cells acquired using Seahorse™ (Fig. A.1).

However, from the biological perspective, microfluidic systems are now being explored to create more physiologically relevant environments for cultured cells. These miniaturized microfluidic devices, often referred to as Organs-on-Chip (OoC) are designed to mimic conditions encountered by cells in whole organs, particularly those of the vasculature [8,9]. They often rely on fluid and gas perfusion combined with 3D tissue cultures and thus are not compatible with assays designed for static 2D cell culture on standard tissue culture or customized plates. For many questions, however, tools that monitor cellular metabolism in real-time would be valuable for understanding complex cellular behaviour under conditions of fluid flow in OoC [10]. In addition, when stem cells or their derivatives are the cellular input for OoC, information can be obtained on how different states of differentiation, maturation, disease or drug treatments impact metabolism [8,9]. Furthermore, metabolic monitoring involves the continuous assessment of relevant culture parameters like oxygen, pH and nutrients. These parameters could be used for rapid optimization of cell culture protocols in OoC, since these devices are typically closed and maintaining cell culture is often affected by the limited availability of oxygen and nutrients. While this can be tuned by altering the flow rate, the current OoC do not have the relevant metabolic readouts to provide the necessary feedback.

Integration of sensing elements inside a microfluidic chip is the first step toward monitoring metabolism *in situ,* and optical chemical- or electrochemical sensors within a microfluidic channel can be used for this. Optical chemical sensors are based on changes in luminescence properties of immobilized dyes in the presence of the analyte; they enable contactless*, in situ,* real-time measurements of metabolically relevant parameters like oxygen consumption rate (OCR), extracellular acidification rate (ECAR) or detect specific metabolites such as glucose and lactate [[11], [12], [13], [14]]. The sensing elements (dye and support matrix) can be miniaturized to fit microfluidic channels allowing seamless integration into the devices without interfering with the tissue [15]. Optical oxygen and pH sensors have already shown versatility within microfluidic chips [[16], [17], [18]]. They have been used to assess the OCR and ECAR of cultured cells inside microfluidic systems and the influence of drugs or nanoparticles on these parameters [10,16]. However, these studies did not compare on-chip data with more commonly used standard assays for assessing metabolic rates, such as the Agilent Seahorse XFe Analyzers™, widely used on static cell cultures.

Here we describe the integration of oxygen and pH sensors into an existing commercial chip and use it to monitor the concentration of dissolved oxygen and pH during cell culture and to measure the OCR and ECAR of human-induced pluripotent stem cells (hiPSCs) during stop-flow measurements. These (non-transformed) cells are highly proliferative, have high metabolic rates and are notable for their reliance on glycolysis [19,20]. We compared metabolic rates obtained under dynamic conditions in the chip with results obtained in the Seahorse system™. On both platforms, we carried out the two most widely used assays for which the Seahorse system was designed: the mitochondrial stress test and the glycolysis stress test. We showed that the on-chip measurements could capture the metabolic activities of the hiPSCs within a microfluidic channel and that the outcome did not differ from experiments performed on the Seahorse system.

## 2 Material and methods

### 2.1 Sensor integration

Liquid sensor formulations were first prepared for both sensors. The oxygen indicator dye platinum (II)meso-tetra(4-fluorophenyl) tetrabenzoporphyrin (Pt-TPTBPF) was synthesized as previously described [12]. Poly-*tert*-butylstyrene particles (ptBS particles) were stained with 2% (w/w) Pt-TPTBPF as described elsewhere [14]. 7% Hydromed D7 (AvanSource biomaterials), a polyurethane-based hydrogel, was prepared in a mixture of ethanol-water (9 ​+ ​1). The Pt-TPTBPF stained particles were mixed in the hydrogel resulting in a 2.5% (w/w) dispersion. The mixture was homogenized by vigorous stirring for 5 ​min.

Preparation of pH sensors was adapted from Müller et al. [16]. Hydromed D4, a polyurethane-based hydrogel, was purchased from AvanSource biomaterials. The aza-BODIPY pH indicator dye 4-(5,5-difluoro-7-(4-hydroxyphenyl)-1,9-diphenyl-5H-5λ4,6λ4-dipyrrolo [1,2-c:2′,1′ f] [1,3,5,2] triazaborinin-3-yl)-N-dodecylbenzamide (compound 5; OHC12) [11] and microcrystalline powder of silanized Egyptian Blue [12] were synthesized in our lab as previously described.

0.1 ​mg aza-BODIPY indicator was dissolved in 100 ​μl THF. This solution and 16.8 ​mg Egyptian Blue were suspended in 415 ​mg 8% Hydromed D4 solution in ethanol-water (9 ​+ ​1). The suspension was homogenized using a sonifier (Branson) with ten pulses at 25% of maximum amplitude for 1-s with a pause of 10s between each pulse. The sensor formulation was used within an hour of preparation.

**Fig. 1:**
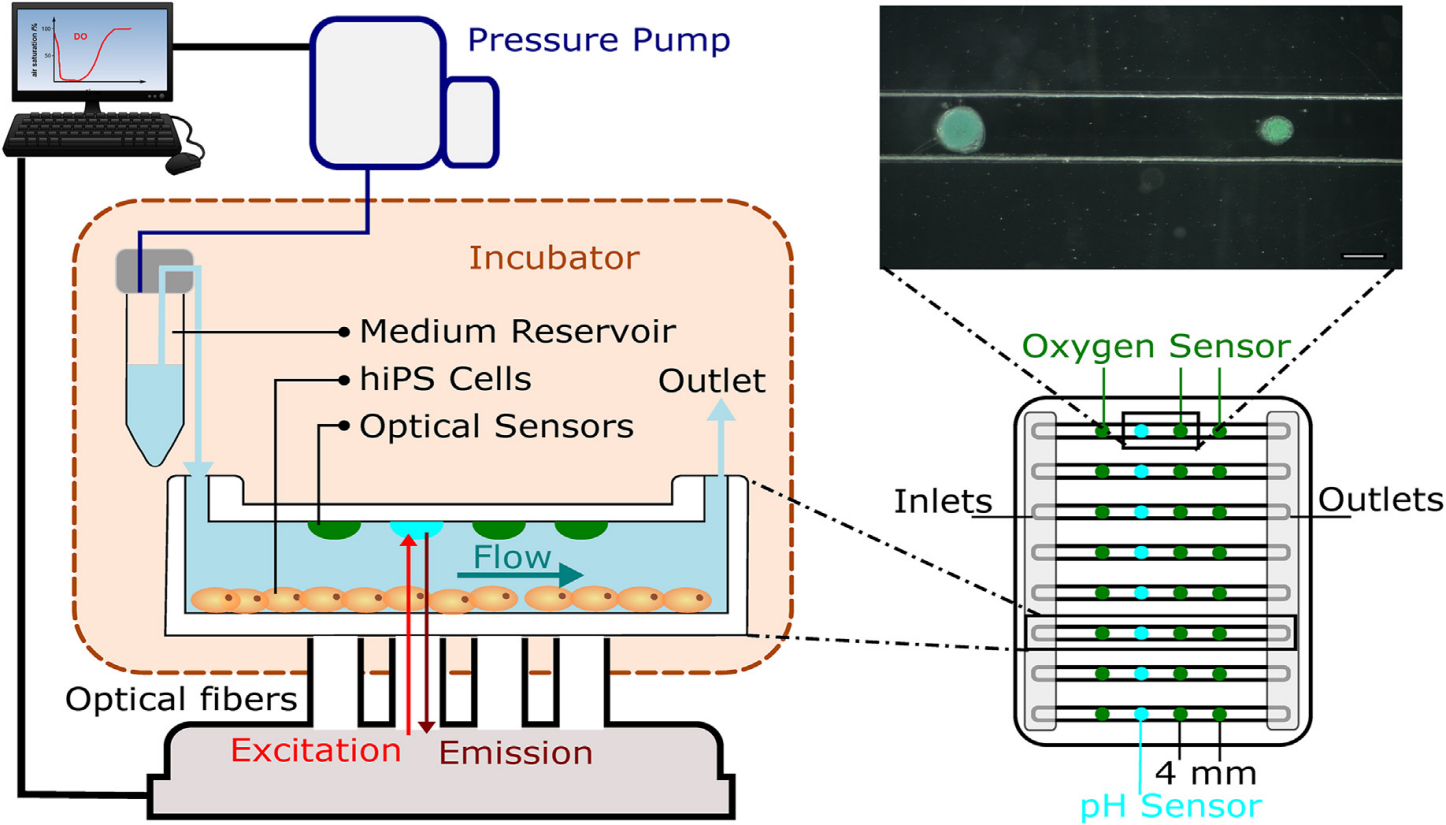
**Schematic design of the experimental setup.** The microfluidic chip features 8 channels with 800 ​μm diameter. Optical oxygen and pH sensors are integrated into the top of the chip, with each channel containing a total of 4 sensors (Scalebar ​= ​500 ​μm). The hiPSCs are cultured on the bottom, and optical read-out of the sensors is performed through the cultured monolayer. The assay is performed inside a tissue-culture incubator, while the pressure pump is kept outside the incubator.

Open Vena8 CGS chips, containing 8 channels with dimensions of 800 ​μm ​(W) x 80 × μm (D) x 28 ​mm ​(L) were purchased from CelliX Ltd. Both sensor formulations were integrated into the microfluidic compartment of the chip via microdispensing. A microdispenser MDS3200+ from VERMES Microdispensing GmbH equipped with a tungsten tappet with a tip diameter of 0.7 ​mm and a nozzle diameter of 200 ​μm was used. The liquid sensor formulation was dispensed in the reservoir, and pressurized air at a pressure of 200 mBar (oxygen) or 400 mBar (pH) was applied. Printing parameters for both oxygen and pH sensors are given in Table 1. Four sensors were integrated into each channel with a distance of 4 ​mm between the centre of the sensor spots. Either three oxygen sensors with one pH sensor in a middle position (Fig. 1) were integrated in one channel or the other way round with three pH sensors and one oxygen sensor. Chips with more oxygen sensors were intended for use in the mitochondrial stress tests, whereas chips with more pH sensors were used for the glycolysis stress test. All sensors were allowed to dry for more than 2 ​h before the chips were sealed with the coverslip provided by CelliX.

### 2.2 Chip preparation

The chips with integrated sensors were sterilized with UV light for 20 ​min in a laminar flow hood. The channels were coated overnight at room temperature with fibronectin in PBS (50 ​μg/ml, bovine plasma, Sigma). This fulfilled two purposes: i) the sensors were hydrated, and ii) the channel was coated with fibronectin to promote cell adherence. Next, the chips were placed in a desiccator and a vacuum applied for 30 ​min to remove air bubbles that formed in the chip overnight. The PBS in the chip was then aspirated, and the chips were used immediately for experiments.

### 2.3 Sensor calibration

The sensor calibration was performed in the same setup as the final experiments. The chip was placed in a custom-made holder in the incubator (37 ​°C, 0% CO_2_) to align 2 ​m polished plastic optical fibers (1/2.2 ​mm) with the integrated sensor spots. 32 optical fibres guided the excitation light and collected the emitted light to the 48-channel phase fluorometer (PyroScience) as a readout [17].

The oxygen sensor material was the same as that described earlier [14]. Two-point calibration at 37 ​°C was performed. Anoxic conditions were induced in one of the channels using 2% sodium sulphite solution. The resulting calibration value was used for all sensors throughout the experiments. Individual calibration of the sensors in air-saturated conditions was performed on the day of the experiment. The oxygen sensor was left to equilibrate in air-saturated PBS or medium at 37 ​°C for the calibration value of the air-saturated conditions. An optical temperature microsensor (PyroScience) was used to compensate for temperature changes.

The pH sensors were calibrated in the same medium used for the assays (see section 2.5). The medium was supplemented with 10 ​mM of MES (2-(*N*-morpholino)ethanesulfonic acid) (Sigma Aldrich) or TRIS (tris(hydroxymethyl)aminomethane) (Sigma Aldrich) and adjusted to 6 different pH values between 5.4 and 9.0 using 1 ​mM NaOH or 1 ​mM HCl at 37 ​°C. The buffers were passed through filters (0.2 ​μm Whatman) to ensure sterility. The buffers were flushed manually through the system and each was allowed to rest for 15 ​min to obtain stable calibration data. The chip was flushed with sterile water before injecting the next buffer. After calibration, the chip was washed with the assay medium. The data obtained were fitted to a Boltzmann equation for calibration (Eq. (1)), where A is the top value, B is the bottom value, pKa is the apparent pK and the slope at the pKa value [16].

(1) $$\cot\left(\delta \varphi \right)=\frac{A-B}{1+10^{\frac{pH-{pK}_{a}}{slope}}}$$

Equation (1)*Boltzmann equation*.

### 2.4 hiPSCs maintenance and cell-seeding

hiPSCs (hPSCReg: LUMCi028-A) were maintained in 12-well tissue culture plates on human recombinant vitronectin-coated substrates and were refreshed daily with E8 medium (ThermoFisher). The hiPSCs were passaged biweekly using 0.5 ​mM EDTA in PBS without Ca^+^ and Mg^+^ (PBS^−^). For cell-seeding in either the microfluidic device or Seahorse plate, cells were washed with PBS^−^ and dissociated using TrypLE 1× (ThermoFisher) and washed in E8 medium containing 0.5× RevitaCell supplement (ThermoFisher). Cells were subsequently counted and seeded at the density specified for each assay.

### 2.5 Quantification of oxidative and glycolytic metabolic capacity measured by Seahorse XF-96 analyzer

hiPSCs were seeded in the Seahorse 96-well plate pre-coated with 10 ​μg/ml fibronectin (Bovine, Sigma) at a density of 70k cells per well in the appropriate pre-made assay medium with the addition of 0.5× RevitaCell. The cells were allowed to recover for a period of 3 ​h before commencing the assay. The assay medium for the mitochondrial stress test consisted of DMEM without bicarbonate (Sigma) supplemented with 5 ​mM glucose (Sigma), 2 ​mM l-glutamine (ThermoFisher), 1 ​mM pyruvate (ThermoFisher), 5 ​mM HEPES (Sigma), and with 0.25% fatty-acid free BSA (Bovogen), followed by pH calibration to 7.4. For the glycolysis stress test assay, the same medium was used without glucose. The mitochondrial stress test was performed by serial injection of oligomycin (1.5 ​μM, Sigma), Carbonyl cyanide-*p*-trifluoromethoxyphenylhydrazone (FCCP; 0.75 ​μM, Sigma), and finally, antimycin A and rotenone together (2 ​μM, Sigma). The glycolysis stress test was performed using injections of glucose (5 ​mM), oligomycin (1.5 ​μM), and finally, 2-deoxy-D-glucose (2-DG; 10 ​mM, Sigma). For both assays, the same conditions and measuring profiles were applied, with a measuring period of 4.5 ​min and a 4 ​min period of mixing or flow between each measurement. The data was normalized by staining live cell nuclei with HOECHST (ThermoFisher) and assessing the total cell number per well.

### 2.6 Quantification of oxidative and glycolytic capacity in a perfusable microchannel chip

The chip was placed in the incubator at 37 ​°C and 0% CO_2_ and allowed to equilibrate for 15 ​min. Cells were subsequently seeded by making a cell suspension of 25 ​× ​10^6^ ​cells/ml in the assay medium with 0.5× RevitaCell and injecting 3 ​μl of the cell suspension into the channel from both sides. The cells were allowed to attach for 30 ​min before adding 12.5 ​μl of assay medium in the reservoirs on one side of the channel, allowing the fresh medium to flow through the channel. Every 30 ​min, 20 ​μl of assay medium was added until 3 ​h after seeding when the assay would commence. The assay was performed by connecting the chip to the tubing using blunt-end Luer lock syringe needles 20G (Techcon). The microfluidic setup consisted of a pressure pump (Fluigent, FLPG Plus) connected to the microfluidic flow control system (Fluigent, LineUP Flow EZ). The controlled flow was connected to a 15 ​ml Falcon (Greiner) tube with a P-CAP (Fluigent) to pressurize the tube. By pressurization, the medium passed via the liquid outlet into the tubing connected to a flow rate sensor (Fluigent, FLOW UNIT size L ±1000 ​μl/min). The pump was controlled from a laptop with the Fluigent A-i-O (v1.0.43) and Fluigent OxyGEN (v1.0.1.0) software. The pump pressure was regulated to maintain a set flow rate over the flow unit of 20 ​μl/min. The flow was subsequently split into 8 channels, resulting in a flow rate of 2.5 ​μl/min per channel. The pressure pump drove the medium from the reservoir and split via an 8-way manifold with additional 85 ​μm resistors (Elveflow), allowing equal flow rates through each channel. The assay was started by perfusing all the channels at the predefined flow rate with the equilibrated assay medium for 7.5 ​min, ensuring that pH and oxygen had reached baseline levels within each channel. Subsequently, four baseline measurements of 4.5 ​min were performed under static conditions, followed by reperfusion of 4 ​min. Subsequent drug exposures were done by disconnecting the chip and perfusing the tubing with the assay medium containing the final drug concentration. The chip was subsequently reconnected, and three measurements were performed according to the same scheme as the basal measurements for each condition.

## 3 Results & discussion

### 3.1 Experimental setup and sensor characterization

**Fig. A2:**
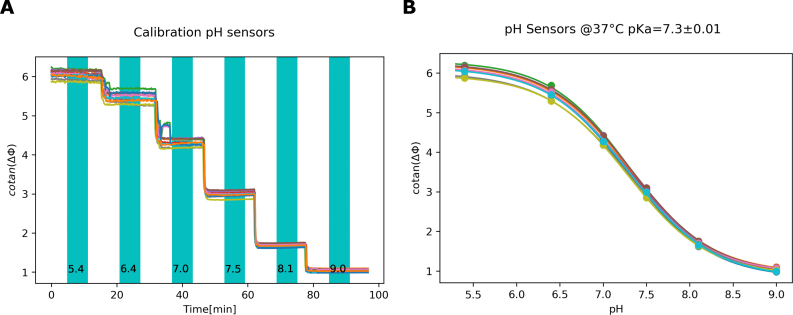
**Representative data of the response and calibration of the** pH **sensors.** A) Response of the pH sensor when exposed to buffered solutions at pH 5.4, 6.4, 7.0, 7.5, 8.1 and 9.0 ​at 37 ​°C. B) Boltzmann fitted calibration curve on the obtained datapoints.

The setup consisted of the Cellix Vena8 microfluidic chip with eight channels. The chip features ports at both ends of the channels. These ports can be used either as reservoirs or connected to a pressure-driven pump, allowing the application of a controlled flow within the system. Each channel featured four sensor spots for either oxygen or pH measurement (Fig. 1). The sensor matrix consists of a biocompatible polyurethane-based hydrogel (Hydromed D4 or D7) doped with either Pt-TPTBPF stained sensor particles for oxygen measurement or Egyptian blue and OHC12 dye for pH sensors. Previous studies have shown that neither sensors are detrimental to cells [16]. Furthermore, the sensors were integrated on the top of the microfluidic channel, whereas the cells were cultured on the bottom, ensuring no direct contact and interaction between the sensors and the cells but still allowing proper measurement of pH and oxygen through the medium. The integrated sensors had diameters of less than 600 ​μm (oxygen) and 700 ​μm (pH) (Fig. 1). The chip was mounted on a custom-made holder aligning the optical fibres for the sensor readout from the bottom. The indicator dyes used were excited with red light and emitted in the near infrared spectrum, allowing sufficient penetration through the cultured cell monolayer and signal generation (data not shown). Furthermore, the measurements were based on luminescence lifetime measurement, which is not affected by inhomogeneities in the light path. Therefore, reliable measurement through the cultured tissue is possible. The oxygen sensors used had been shown to remain stable during cell culture at 37 ​°C in previous studies [10,27,28]. The pH sensors showed a reproducible response to pH with an apparent pKa (point of inflexion of calibration curve) of 7.30 ​± ​0.01 and a resolution of 0.004 ​at 37 ​°C after the six-point calibration (Fig. A.2). The dynamic range of the sensor was from pH 6.3 to pH 8.3, which is a suitable measurement range for the tissue culture conditions from pH 6.3 to 7.5. The response time (t95) was approximately 50 ​s for a change from pH 7.0 to 7.5 via manual flushing of the system (Fig. A.2), showing a rapid response time in the relevant range. The long-term stability of the pH-sensor was not assessed as it showed little drift over 7 days in previous studies [16].

### 3.2 Optimization of cell-seeding using pH-sensors to monitor tissue-culture acidification

**Fig. 2:**
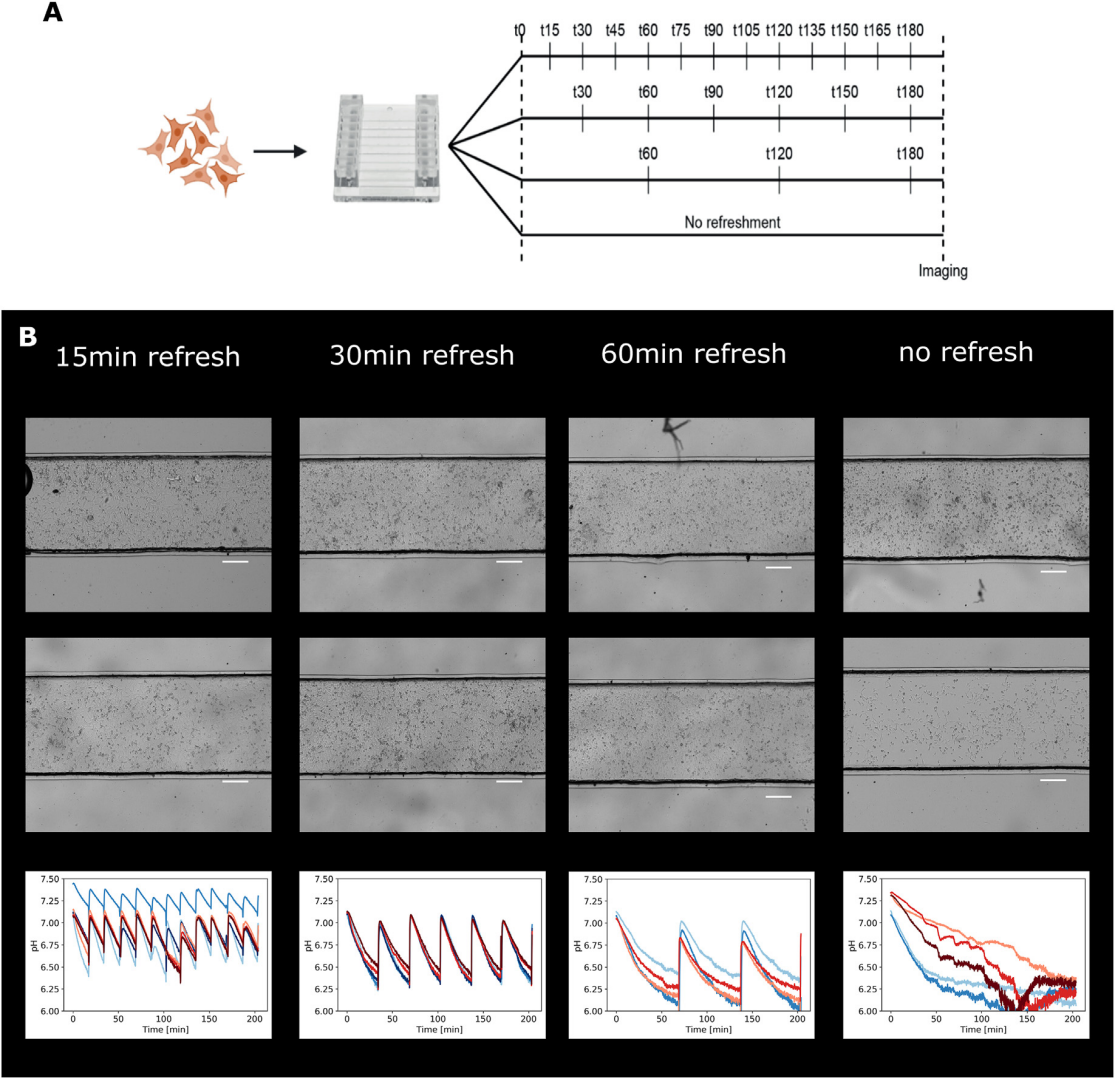
**Optimisation of seeding conditions using the sensors.** A) During the attachment of the cells, the medium was refreshed by adding 12.5 ​μl medium at 15-, 30-, and 60-min intervals. In an additional control channel, the medium was not refreshed. B) The effect on the cells was investigated using microscopy and the read-out of the pH sensors. Each panel shows duplicate images acquired at the end of the experiment and the read-out of the pH measurements. Scale-bar ​= ​250 ​μm.

After the cells were seeded, monitoring of dissolved oxygen concentration and pH of the medium was started directly using the integrated sensors. Cell attachment took up to 3 ​h, during which the microfluidic pumps were not connected to the chip and there was no flow in the channel. pH levels dropped drastically during this period, to less than pH 6.2, outside the measurement range. This acidification of the medium led to cell death and prevented proper formation of a cell monolayer. Therefore, gentle flow was introduced at different time intervals by adding 12.5 ​μl of medium to one of the reservoirs. Time intervals of 15, 30, and 60 ​min were tested, during which the sensor readout was followed to find the best culture conditions (Fig. 2). After 3 ​h, the cells were examined using bright-field microscopy (Fig. 2B). As before, pH levels dropped to less than pH 6.2 without medium refreshment leading to variable cell confluence. Medium refreshment every 60 ​min also led to a drop in pH below 6.2. However, in samples refreshed every 15 or 30 ​min, the pH did not drop below 6.2. The pH difference was greater with refreshment intervals of 30 ​min rather than 15 ​min (ΔpH 0.75 ​± ​0.02 and 0.40 ​± ​0.07, respectively) but refreshment every 15 ​min reduced the cell number in the centre of the channel, possibly because cells were disrupted during attachment and were then washed away. Only with refreshment intervals of 30 or 60 ​min were the cells able to form a confluent monolayer. However, the pH dropped below 6.2 in the 60 ​min interval preventing accurate measurements. Thus, the 30 ​min interval was chosen for further experiments.

### 3.3 Assessing oxygen consumption rate (OCR) and extracellular acidification rate (ECAR) by measuring oxygen and pH

To measure OCR and ECAR accurately during the assays within the chip, the same measuring protocol was used as within the Seahorse. The Seahorse system measures by lowering a probe into each of the wells, creating a tightly sealed micro-well of 1.6 ​μl. The smaller volume of the micro-well ensures more rapid depletion of dissolved oxygen and acidification of the medium, which in turn allows accurate assessment of the metabolic rates. The oxygen tension and pH measurements are made every 16 ​s for a total of 4.5 ​min, after which the measurements stop. Subsequently, the airtight seal created by the probe is broken, mixing the medium and returning oxygen and pH levels to baseline. After this mixing period, the probe is again lowered into position and measurements are resumed. Each of these measuring periods is used to generate a single OCR and ECAR datapoint by fitting a first-order polynomial and taking the derivative.

**Fig. 3:**
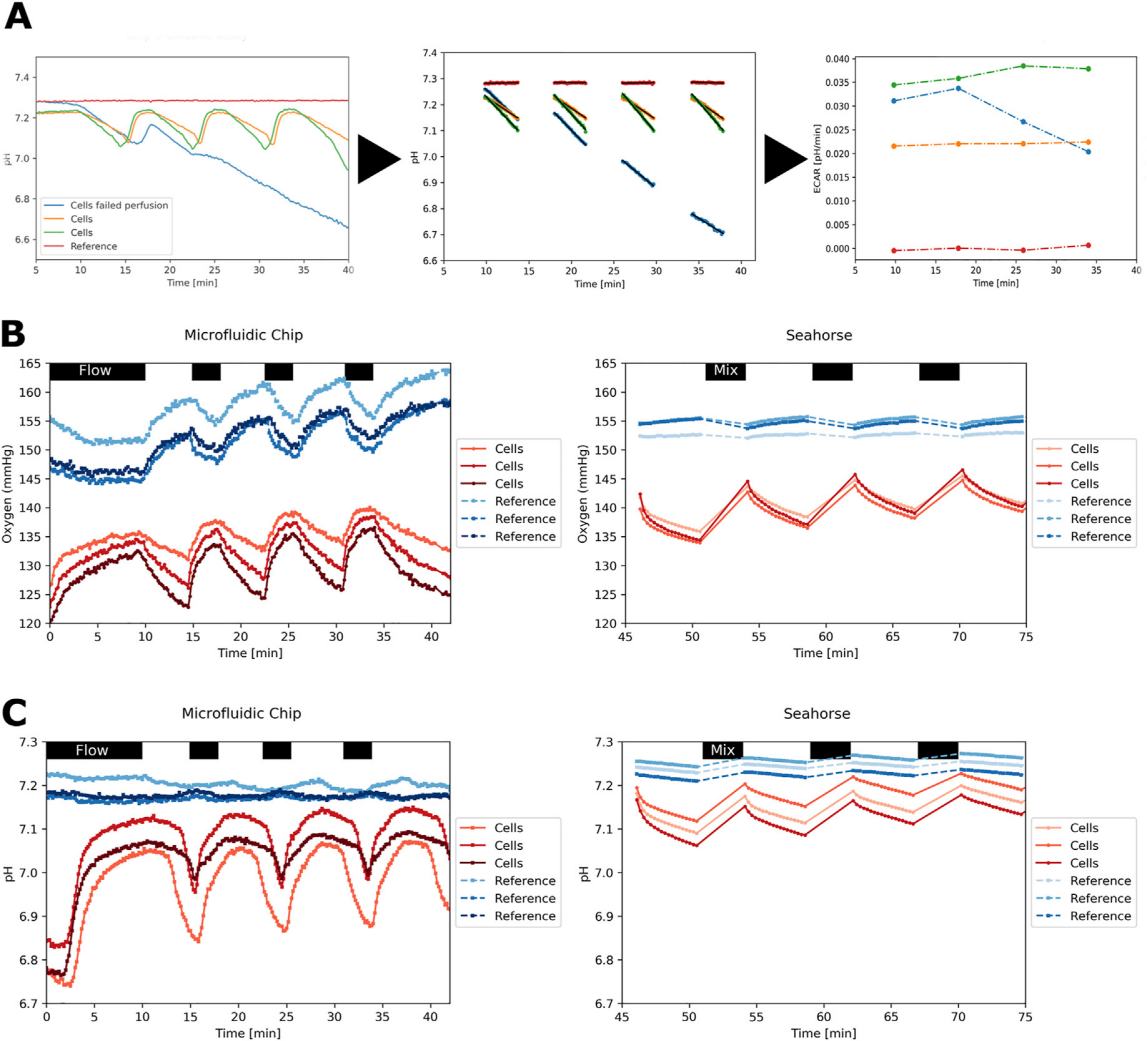
**Derivation of ECAR and raw data comparison from chip and Seahorse.** A) On the left, complete data acquisition during the stop-flow protocol. The colour-to-data assignment shown in this panel also applies to the following panels. The middle panel show the isolated periods without flow and the applied polynomal fits (black lines) from which the extracellular acidification rate (ECAR) can be calculated, plotted in the right-side panel. B & C) Comparison of Raw Data of measurements in the chip (left) and the seahorse system (right) during baseline measurement for mitochondrial assay (B) and glycolysis assay (C). Flow/Mixing intervals are marked in black. The measurement interval of 4.5 ​min shows changes in oxygen partial pressure and a decrease of pH in the chip and the Seahorse for measurements with cells. References are empty channels without cells.

**Fig. A3:**
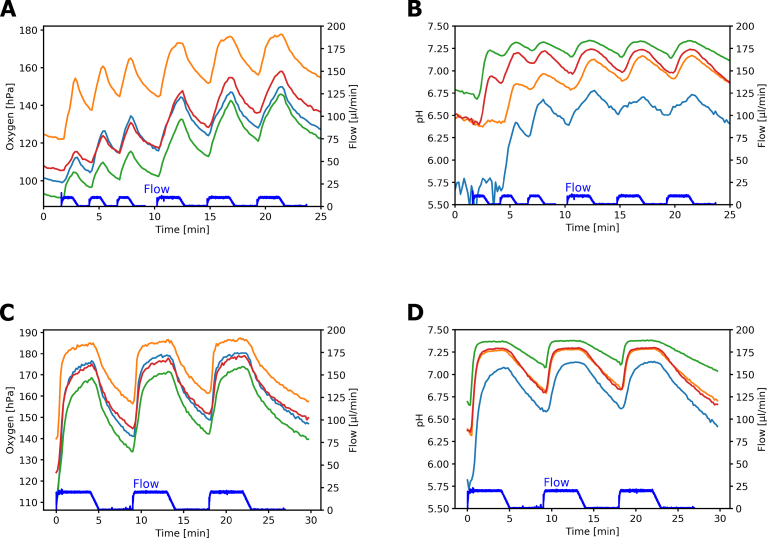
**Optimisation of the microfluidic pump interval on the chip.** A-D) Measurements of oxygen depletion and resaturation within the microfluidic channels (A, C) and pH measurements showing the acidification and recovery (B, D) during different flow intervals. The blue line shows 3× flow intervals of 1 ​min on/off, followed by 2 ​min on/off with flow rates of 10 ​μl/min (A, B), and stop-flow interval of 5 ​min on/off (C, D). The left side y-axis denotes the measured analyte, while the right side y-axis denotes the flow rate.

To assess whether we could apply the same principle to measurements acquired from the sensors on our chip, optimal timing for the flow- and static cultures were first determined (Fig A3). Perfusion intervals of 1–5 ​min were tested to determine which timespan was sufficient to reoxygenate the channel and reset the medium pH back to baseline. The period for static conditions was optimized from 1 to 10 ​min to allow an analogous drop in oxygen and pH as in the Seahorse. OCR and ECAR were derived by analyzing the data during periods of static culture, fitting a first-order polynomial and taking the derivative. Fig. 3A demonstrates the assessment of ECAR by comparing one background channel without cells to three channels containing an equal number of cells. ECAR measurements remained stable during this period, while even unintentionally failed reperfusion allowed estimation of ECAR. However, failed reperfusion led to a decrease in ECAR over time; therefore, these data were excluded from further evaluation. Using this method to determine the ECAR, a stop-flow protocol was designed to mimic the Seahorse protocol, where 4.5 ​min measurements under static conditions were performed. These measurements were followed by 4 ​min of reperfusion to allow oxygen and pH to reset to baseline values. We verified that this yielded similar results to a Seahorse experiment with identical settings for measurement and mixing. Fig. 3B shows oxygen depletion from the medium is linear in our chip, like that observed in the Seahorse. Oxygen depletion during the measurement phase in the chip is approximately 10 ​mmHg, while the Seahorse showed depletion of approximately 5 ​mmHg in the same time period. Seahorse does not continue measurements during mixing, while our chip setup allows continuous monitoring, even during perfusion. We found an increase in oxygen concentration in our reference channel and the Seahorse system blank wells during the static periods. There are several possible explanations – or a combination of them – for this phenomenon. One is that the medium is not entirely equilibrated with the ambient conditions in the incubator. This could involve slightly too low temperature due to the large temperature capacity of the medium and insufficient pre-heating. An increase in the temperature of the medium in the channel would lead to an elevated signal from the oxygen sensor while the actual oxygen concentration within the medium remains the same. Additionally, a slight difference in the oxygen concentration in the media and the ambient air in the incubator could lead to re-equilibration during the flow stop. Another possibility could be the oxygen storage capacity of the thermoplastic chip material. When thermoplastics are heated, they release some of their stored oxygen, which also increases the oxygen concentration in the channel. All of these effects would presumably also be present in the channels with cells but not evident as the cells consume more oxygen than the increase in the reference channels showed. Before further evaluation, the reference measurement was used as baseline correction for the cell measurements, thereby eliminating the measuring error induced by the observed effect.

ECAR measurements in the chip also resembled the Seahorse (Fig. 3C). The measurement showed a significant decrease in the pH of 0.15 during the measurement time, while Seahorse showed a smaller decrease in pH of 0.1. Here we found that the reference channels in the chip and the background wells in the Seahorse showed no significant differences in pH during the measurement or flow periods. Overall, we obtained outcomes from sensors in the chip which resembled the Seahorse assay closely. We note that the reference channel values are important to correct measurements in the channels containing cells.

### 3.4 Mitochondrial respiration and glycolytic acidification can be assessed by on-chip monitoring of pH and oxygen

**Fig. 4:**
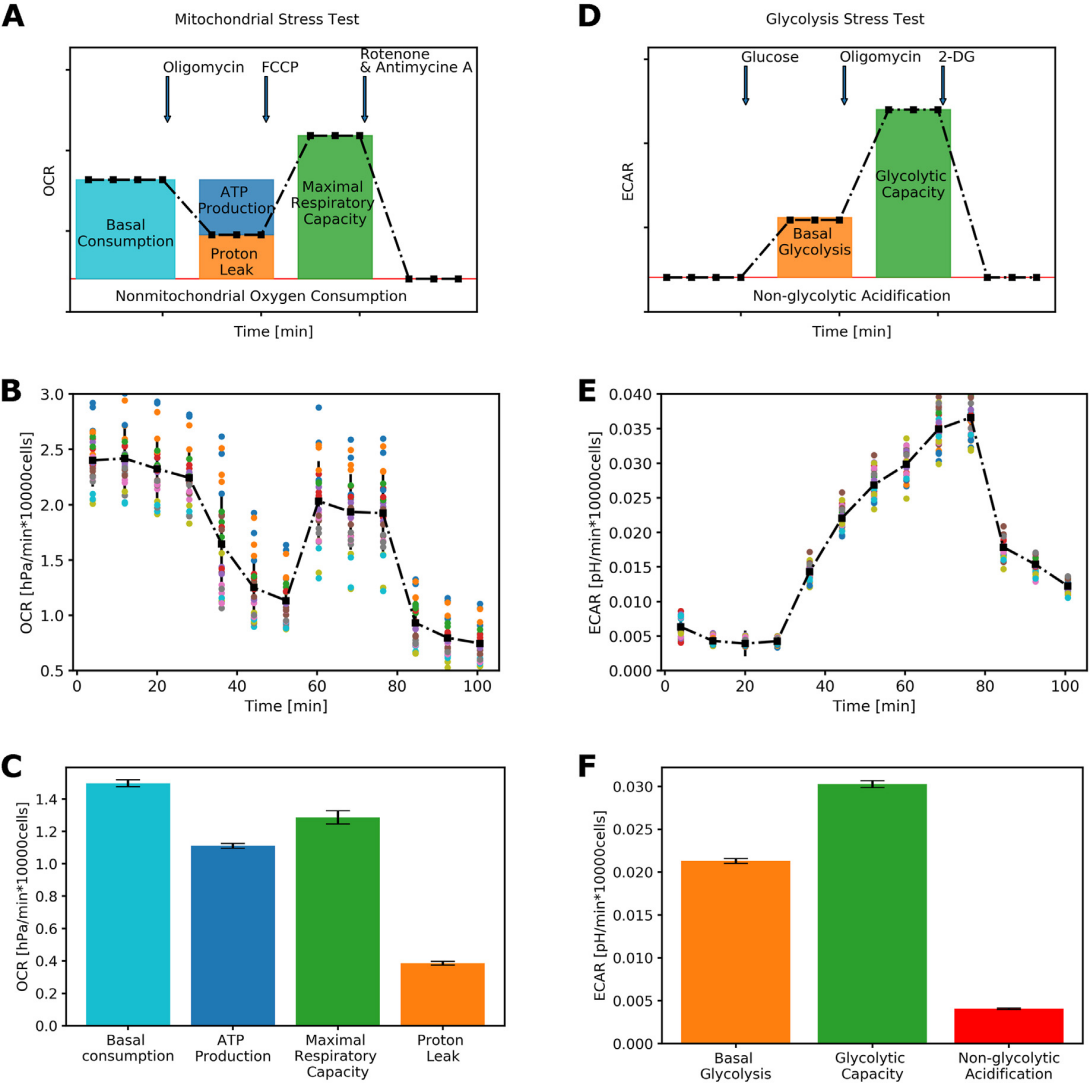
**Representative Seahorse experiment and data.** A-C) The mitochondrial stress test, with a graphic representation (A), representative data obtained from hiPSCs (B), and the quantification of the data (C). D-F) Glycolytic stress test, first depicted as a graphical representation (D), a representative experiment with hiPSCs (E), and final quantification from the experiment (F). Data mean ​± ​standard deviation, with 28–30 replicates per condition and a single experiment. Oxygen consumption rate (OCR) is defined as the depletion of oxygen (hPA) per minute, normalised by total cell count. Extracellular acidification rate is the decrease in pH per minute, normalised by total cell count. Arrows note the moment when a drug compound is injected into the plate.

The mitochondrial- and glycolysis stress test assays were performed on the chips and the Seahorse. These tests are the most commonly used in the Seahorse system and allow assessment of the metabolic capacity of cells to generate ATP via glycolysis or oxidative phosphorylation (OXPHOS) (Fig. 4).

The mitochondrial stress test was performed by serial injection of oligomycin (1.5 ​μM), blocking OXPHOS complex V and allowing quantification of OCR dedicated to ATP production, followed by FCCP (0.75 ​μM) injection, uncoupling the inner-mitochondrial membrane and assessing maximal respiration capacity. A final injection of antimycin A and rotenone (both 2 ​μM), blocking complex III and complex I, respectively, allows quantification of the proton leak and non-mitochondrial oxygen consumption (Fig. 4A). Performing this assay in the Seahorse on hiPSCs results in an expected profile, with both the injection of oligomycin and antimycin A with rotenone resulting in a strong decrease in OCR, while the injection of FCCP leads to an increase in OCR (Fig. 4 B). Calculating the contribution of OCR in the hiPSCs under the various conditions revealed that the contribution of OCR towards ATP production is lower than the basal consumption, caused by a high proton leak (Fig. 4C). Proton leak is the result of protons leaking over the inner membrane of the mitochondria, where they react with oxygen to generate H_2_O and release further energy in the form of heat. This process does not contribute to the charging of ADP into ATP and is, therefore, inefficient. Additionally, injection of FCCP did not increase the OCR above the basal levels, thus suggesting that the OXPHOS capacity of the hiPSCs is already used at full potential under basal conditions [[29], [30], [31]]. These results match with previously published work and show that hiPSCs predominantly rely on glycolysis for their metabolic and energetic needs [19,20]. The glycolysis stress test (Fig. 4D) was performed by basal measurements in a medium containing no glucose, the first measurements set a baseline, assessing non-glycolytic ECAR. Glucose (5 ​mM) was subsequently added to the medium, restarting glycolysis in the cells and allowing a measure of the basal level of glucose utilization via glycolysis. Subsequently, oligomycin (1.5 ​μM) was added, blocking ATP production via OXPHOS, and forcing the cells to compensate by upregulating glycolysis; this results in the maximal glycolytic rate of the cells. Finally, 2-DG (10 ​mM) was added, which acts as a competitive inhibitor of the enzyme phosphoglucoisomerase, blocking glycolysis in the second step. This drug was used to verify the absence of glycolysis in the first measurements of the assay. Performing this assay in the Seahorse on hiPSCs resulted in the expected profile, with the addition of glucose and oligomycin leading to a significant, approximately 7-fold, increase in ECAR (Fig. 4E and F). Quantification showed that both the injection of glucose and oligomycin contributed to increases in ECAR, revealing that hiPSCs have additional (spare) glycolytic capacity, which is not used during normal basal conditions [19,20,32].

**Fig. 5:**
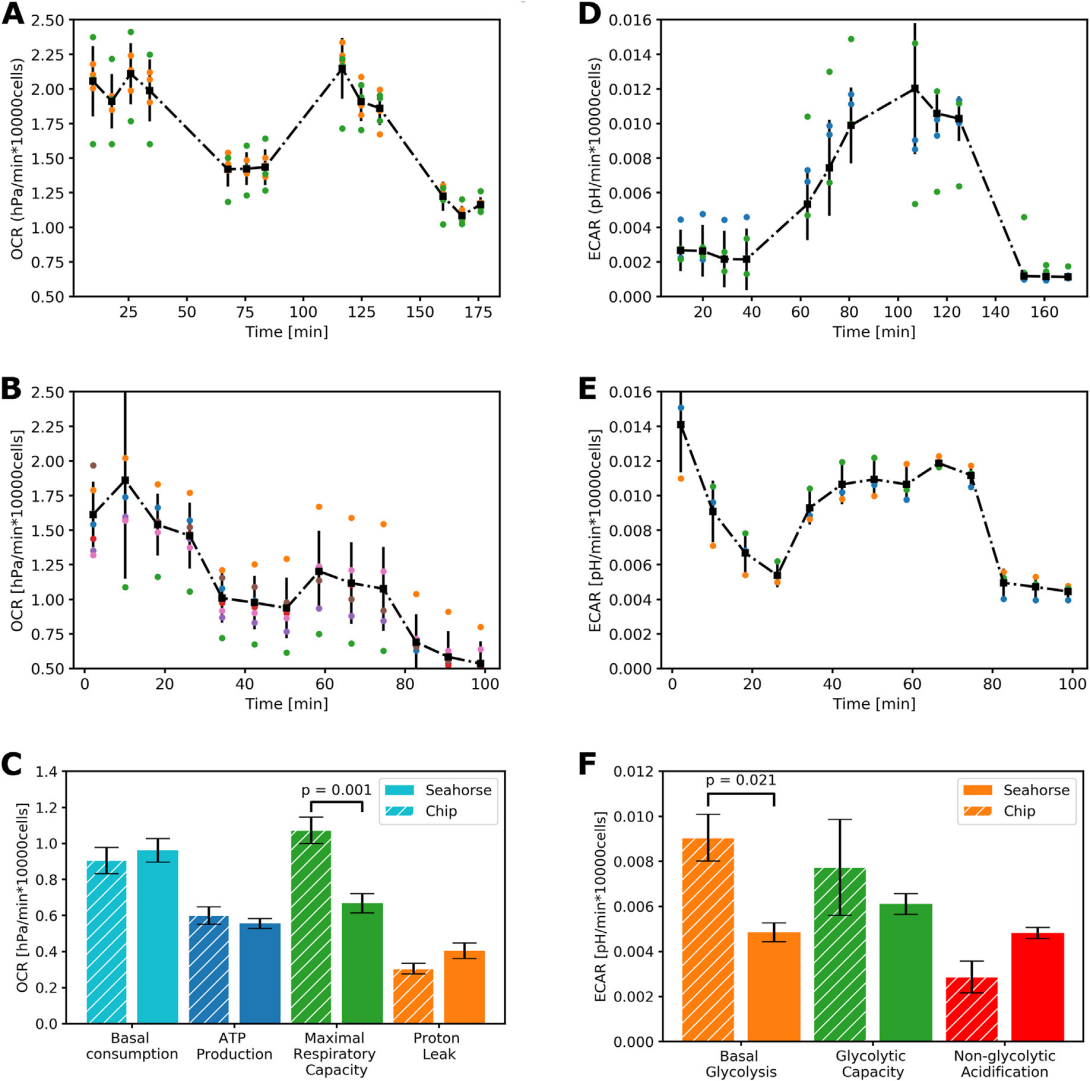
**Comparison between measurements on-chip and in the Seahorse system.** A-C) The mitochondrial stress test assay with the chip (A, n ​= ​6) is compared to the Seahorse (B, n ​= ​7) and quantification of different respiratory states (C). D-F) The glycolytic stress test assay on the chip (D n ​= ​4), compared to the Seahorse (E) and the quantification of the glycolytic capacity (F, n ​= ​3), with the quantification of the glycolytic activity. All data represent mean ​± ​standard deviation.

We performed the assays in parallel on both Seahorse and the chip to assess whether the sensors on the chip could replicate the measurements obtained by Seahorse on the same batch of cells and medium and on the same day. Cells in both the Seahorse and chip assay were treated with the same final concentrations of the drugs, where the Seahorse made use of a 10× concentrated injection while the chips were perfused with the medium containing the compound at the final concentration. Fig. 5 A and B show the OCR measured in the chip and the Seahorse during the mitochondrial stress test assay, respectively. It is evident that the OCR was similar between the two systems after correcting for total cell count. Additionally, the expected effects of adding the drugs were observed in both assays. Quantification of the assay showed no significant differences between the two systems for the OCR at basal respiration, ATP production and proton leak, while maximal respiration was increased on the chip (Fig. 5C). The time course in Fig. 5D and E shows the kinetics during the glycolysis stress test in both the chip and the Seahorse. Like the mitochondrial stress test, the measured flux on the chip resembled the traces observed in the Seahorse system, with quantification showing no significant difference between non-glycolytic acidification and glycolytic capacity and an increase in basal glycolysis on the chip (Fig. 5F). Of note is the relatively low glycolytic capacity observed in these experiments, which were similar to the basal glycolysis in both the chip and the Seahorse. These results suggested that the hiPSCs use their maximal ATP production rate under the culture conditions present, in contrast to our Seahorse results initially obtained which showed a large glycolytic capacity. However, the hiPSCs demonstrated this behaviour in both platforms, indicating it is not an inaccuracy of the measuring method. Differences in cellular phenotype were, however, observed between the methodologies. The hiPSCs plated in the Seahorse displayed a lower maximal respiratory capacity and basal glycolytic rate. With the similarities in absolute values of OCR and ECAR, we estimate that the differences are most likely to be explained by differences in tissue-culture plate, plastic versus glass and the additional administration of medium perfusion. A previous study has indicated transcriptional differences between hiPSCs derived in tissue-culture plates when compared to hiPSCs derived in a microfluidic device [33]. To better understand these differences, more metabolic studies are needed, comparing different culture methods and including perfusion to decipher the contribution of methods used to a difference in metabolic phenotype.“

### 3.5 Comparison with existing systems

Recent studies have used either optical or electrochemical sensors to assess cellular metabolism inside microfluidic systems (Table 1). Most of these systems allow active perfusion of the cultured tissues and continuous refreshment of the culture medium. Some systems incorporated advanced 3D cell culture techniques, illustrating their ability to capture greater biological relevance than conventional 2D culture on plastic plates. Whilst the study here used 2D cell culture, the sensors have already been combined with 3D cell culture constructs [10]. Together, this shows flexibility for incorporating and accurately assessing more complex cultures under fluidic flow than in standard assays such as Seahorse.

**Table 1:** Overview of previous publications using sensors for assessing metabolic flux.

System	Sensors	Perfusion	Tissue type	Platform	Throughput	Sensing principle	Sensor-tissue interaction	Intervention/medium exchange	Source
This work	Oxygen/pH	yes	2D	microfluidic chip	8-channels	Optical	no	yes/yes	This paper
Seahorse XF analyzer	Oxygen/pH	no	2D	self-contained	96-well	Optical	no	yes/no (treatment injection)	Ferrick et al., 2008 [6]
MitoXpress	Oxygen/pH	no	2D	plate-reader/microscope	any well-plate	Optical	yes (for intra-cellular measurements)	no/no	Hynes et al., 2009 [7]
HepaChip-MP	Oxygen	yes	Sinusoid	microfluidic chip	24 well	Optical	no	yes/yes	Busche et al., 2022 [17]
Dornhof 2022	Oxygen/Glucose/Lactate	yes	Spheroids/3D	2-gel compartments	2 compartments	Electrochemical	yes (Sensors consume oxygen)	yes/yes	Dornhof et al., 2022 [21]
Tanumihardja 2021	Oxygen/pH	no	2D	Chip	single-well	Electrochemical	yes (Sensors consume oxygen)	yes/no (treatment injection)	Tanumihardja et al., 2021 [22]
Liang 2019	pH	yes	2D	Microfluidic chip	single	Electrochemical	yes (Sensors consume oxygen)	yes/yes	Liang et al., 2019 [23]
Obeidat 2019	Oxygen/pH/Glucose/Lactate	no	2D/3D very small	Single-well chip	single	Electrochemical	yes (Sensors consume oxygen)	yes/no (treatment injection)	Obeidat et al., 2019 [24]
Weltin, 2017	Oxygen/Lactate	no	3D	Electrical probe	96-well plate	Electrochemical	yes (Sensors consume oxygen)	yes/no (treatment injection)	Weltin et al., 2017 [25]
Bavli 2016	Oxygen/Glucose/Lactate	yes	Bioreactor	Reactor-on-chip	9 samples	Optical/Electrochemical	no	yes/yes	Bavli et al., 2016 [26]
Rennert, 2015	Oxygen	yes	2D	Microfluidic chip	9-channel	Optical	no	yes/yes	Rennert et al., 2015 [18]

Some sensor types used in commercial and custom-made systems interfere with the cells, making it challenging to assess cellular metabolism accurately. Intracellular sensors can directly interfere with the cells by sequestering intra-cellular oxygen, while electrochemical oxygen sensors actively consume oxygen while in operation, directly influencing the cell culture conditions and contributing to the depletion of oxygen. Within the small medium volumes often used in OoC-tissue culture, this oxygen consumption can directly impact the measurements and is, therefore, less reliable in assessing cellular metabolic flux (Table 1).

One of the significant advantages of the Seahorse system is the ability to carry out assays in 96-well plates in parallel. This scalability has made the system favoured for high-throughput data acquisition, important for rapid screening, statistical significance and, more importantly, biological relevance. Most published microfluidic systems do not allow easy scaling, which is increasingly required for OoC systems. The system here showed 8 microfluidic channels and 32 sensors in parallel. The readout device supports 48 sensors which, in principle, enables scale-up to 12 assays in parallel.

## 4 Conclusion and outlook

In this study, we described a microfluidic chip with integrated optical oxygen and pH sensors that allowed monitoring of culture conditions and assessment of metabolic rates of cells with outcomes similar to that of standard assays in the Seahorse. We used hiPSCs as an exemplar to compare these systems directly since they are not transformed yet and have a relatively high metabolic rate. One of the challenges of OoC is the small culture volumes used in the microfluidic channels; the volume of culture medium available to the cells is much lower than in classical cell culture. A direct consequence is a more rapid depletion of oxygen and nutrients, the build-up of waste products and the changes in osmolarity due to evaporation and medium acidification, all of which can be detrimental to cells within OoC. Constant monitoring of pH and oxygen allowed us to monitor these medium changes during the initial setup of the microfluidic devices, providing essential information for optimizing protocols and ensuring consistent experimental conditions between repeated experiments. While initially challenged by inconsistent seeding outcomes within the chip, pH monitoring allowed us to identify an optimal protocol to create a consistent confluent monolayer of cells in the microfluidic channel.

In this work, we showed that optical sensors can accurately assess the metabolic flux of cells within a microfluidic device. Similar sensors have been used previously, which showed applications in revealing aspects of the biological responses of cells within OoC environments. However, none of the previous studies compared the outcome of metabolic assays in the cells head-to-head with more standard assays on the same cells. We assessed the metabolic rates of the cultured cells during stop-flow measurements and found that chip results were similar to the static measurements performed in the Seahorse. The mitochondrial- and glycolysis stress tests were also performed on both the chips and the Seahorse. We found very similar OCR and ECAR values in both systems, showing that the sensors can be reliably used in chips to assess ATP production rates from OXPHOS and glycolysis.

In sum, this study shows that our approach to combine optical sensors and stop-flow measurements can be used in microfluidic devices to obtain *in situ* data of similar relevance as the Seahorse system, with the added value that the cells can be cultured under physiological conditions with flow before performing the assay. This approach can be transferred to other microfluidic systems and OoC, as shown previously. This study sets the stage for follow-up studies using a multiplicity of differentiated hiPSCs as mono-cultures or multicell type combinations, and continuous quantification of metabolic activity in complex microfluidic cell culture devices.

## Credit author statement

**Stefanie Fuchs:** Conceptualization, Methodology - sensors, Investigation, Formal analysis, Visualization, Writing-original draft & editing. **Ruben W.J. van Helden:** Conceptualization, Methodology – cell culture, Investigation, Formal analysis, Visualization, Writing-original draft & editing. **Maury Wiendels:** Methodology, Investigation. **Mees N.S. de Graaf:** Methodology. **Valeria V. Orlova:** Supervision, Funding acquisition, Writing - review & editing. **Christine L. Mummery:** Supervision, Funding acquisition, Writing - review & editing. **Berend J. van Meer:** Conceptualization, Supervision, Writing - review & editing. **Torsten Mayr:** Conceptualization, Supervision, Funding acquisition, Writing - review & editing.

## Declaration of competing interest

The authors declare the following financial interests/personal relationships which may be considered as potential competing interests: Torsten Mayr reports a relationship with PyroScience GmbH that includes: employment and equity or stocks. Christine Mummery reports a relationship with Ncardia Netherlands that includes: board membership and equity or stocks.

## Data Availability

Data will be made available on request.
