# Supplementary material for: On-chip analysis of glycolysis and mitochondrial respiration in human induced pluripotent stem cells
Source: Mater Today Bio. 2022 Oct 31;17:100475. doi: 10.1016/j.mtbio.2022.100475 (PMC9647220; doi:10.1016/j.mtbio.2022.100475)
Supplement: Multimedia component 1 [file mmc1.pdf]

Table A 1: Microdispenser settings

	Oxygen sensor	pH sensor
Tappet lift [%]	35	75
Rising time [ms]	0.2	0.3
Opening time [ms]	0.1	0.1
Falling time [ms]	0.15	0.1
Delay [ms]	0.1	0.1
Number of pulses	3	4
Number of repetitions	5	10
